# The effect of narcotics on ferroptosis-related molecular mechanisms and signalling pathways

**DOI:** 10.3389/fphar.2022.1020447

**Published:** 2022-10-13

**Authors:** Xiaoqin Zeng, Jingda Li, Fuyuan Yang, Rui Xia

**Affiliations:** ^1^ Department of Anaesthesiology, The First Affiliated Hospital of Yangtze University, Jingzhou, Hubei, China; ^2^ College of Life Sciences, Yangtze University, Jingzhou, Hubei, China; ^3^ School of Basic Medicine, Yangtze University Health Science Center, Jingzhou, Hubei, China

**Keywords:** ferroptosis, anaesthetic, iron overload, glutathione peroxidase 4, cystine glutamate transporter

## Abstract

Ferroptosis is a novel programmed cell death form characterized by iron-mediated reactive oxygen species-induced lipid peroxidation and subsequent cell damage that is distinct from apoptosis, necroptosis, pyroptosis, and autophagy. Most studies on ferroptosis are based on its function and mechanism, but there have been relatively few studies on the effects of drugs, especially anaesthetics, on ferroptosis. Therefore, we summarized the recent literature on the effects of anaesthetics on ferroptosis to understand the underlying mechanism. In particular, we focused on the targets of various anaesthetics in different mechanisms of ferroptosis and the effects of ferroptosis induction or inhibition by narcotics on various diseases. The aims of this review are to provide a relatively reasonable drug regimen for clinicians, to explore potential ferroptosis protection drugs and targets, to reduce perioperative complications and to improve the postoperative performance of patients, especially those who are critically ill.

## Introduction

In 2012, a new regulatory cell death (RCD) method was first proposed and named ferroptosis (Dixon et al., 2012). Since then, a series of studies have focused on ferroptosis. Ferroptosis is a novel programmed cell death form driven by iron-mediated reactive oxygen species (ROS)-induced lipid peroxidation and subsequent cell damage, which is different from other RCDs, such as apoptosis, necroptosis, pyroptosis, and autophagy at biochemical, morphological and genetic levels (Bergsbaken et al., 2009; Linkermann et al., 2014; Skommer et al., 2010; Yang and Stockwell, 2008; Zhang et al., 2015) (Table 1). At the subcellular and cellular levels, ferroptotic cells under the influence of anaesthetics exhibit a characteristic round morphology prior to cell death, resembling necroptotic cells, but without organelle swelling or plasma membrane permeabilization (Dixon et al., 2012; Wu et al., 2020). In ferroptotic cells, the nuclear structure is intact, and there is no chromatin condensation and rupture, nuclear pyknosis rupture, nucleolus disappearance or apoptotic body formation, which are characteristic features of apoptosis (Liang et al., 2019). Furthermore, morphological features such as intense membrane blebbing and loss of plasma membrane integrity in pyroptosis and double-membrane wrapping, autophagolysosome formation from autophagic cells were not observed in ferroptotic cells (Liang et al., 2019). The only unique morphological features of ferroptosis are mitochondrial shrinkage, reduction or disappearance of mitochondrial cristae, and increased bilayer density (Yagoda et al., 2007; Zhao et al., 2022). Although different types of mechanisms regulating cell death have different morphological and biochemical characteristics, there is still some interference between regulators and the components of these different processes. For example, the degradation of ferritin by autophagy can promote ferroptosis (Ma et al., 2016). By observing the apoptosis induced by Tumour necrosis factor related apoptosis-inducing ligand (TRAIL), it can be confirmed that the combination of ferroptosis inducers and TRAIL can significantly improve the killing effect on tumour cells (Lee et al., 2018).

**TABLE 1:** The main morphological and biochemical features, key genes, inducers and inhibitors of ferroptosis, apoptosis, autophagy, necroptosis, and pyroptosis. And the effect of anaesthetics on cell death.

Cell death	Ferroptosis Yang and Stockwell (2008)	Apoptosis Skommer et al. (2010)	Autophagy Zhang et al. (2015)	Necroptosis Linkermann et al. (2014)	Pyroptosis Bergsbaken et al. (2009)
Iconic feature	Mitochondrial cristae decrease or disappear, mitochondrial outer membrane ruptures and shrinks, mitochondria shrink under electron microscope, and the density of double membrane increases	Chromatin condensation breakage, nucleolus disappears, nucleus pyknosis, ruptures, autophagosome formation	Autophagy lysosome formation	Plasma membrane permeabilization, swelling of organelles (e.g., mitochondrial)	Inflammasome formation
Other features	Iron-dependent nucleus without rupture, cell membrane rupture	Cell integrity, cell shrinkage, cytoplasmic efflux, membrane vacuolization	No change in nucleus, no change in cell membrane	Cell membrane rupture, cytoplasm and nucleus disintegrate	Cells are swollen, membranes are blistered, DNA is broken, but the nucleus is intact
Biochemical features	Iron and ROS accumulation, lipid peroxidation exaltation, cystine uptake decreased, GSH reduced	Cytochrome c released, activation of caspases, intracellular calcium increased	LC3-I to LC3-II conversion	Activation of RIP1, RIP3 and MLKL, drop in ATP levels, RARP1 hyperactivation	Caspase-1 and caspase-7 activation, IL-1β and IL-18 secretion
Key genes	GPX4, LSH, TFR1, Nrf2, xCT	Caspase, P53, Fas, Bcl-2, Bax	ATG5, ATG7, DRAM3, TFEB	LEF1, RIP1, RIP3	Caspase-1, IL-1β, IL-18
Inducers	Erastin, RSL3, Sorafenib, RAS, p53	P53, Bax, Bak, TGF-B	ATG5, ATG7, other ATG family proteins, Beclin1	TNFα, Z-VAD-FMK, PAMPS	ZnO-NPS, Ivermectin
Inhibitors	GPX4, SLC7A11, Nrf2, FSP1, Ferrostatin-1, Liproxstatin-1, DFO	Bcl-2, Bcl-XL, Z-VAD-FMK, IL-4	3-Methyladenine, mTOR, Wortmannin, Spautin1, PIK-III, Compound 31	Necrostatins (e.g. Nec-1)	Necrosulfonamide
Promotes cell death under the influence of anaesthetics	Ketamine, bupivacaine, and propofol, *etc.* promote ferroptosis in cancer cells. Sevoflurane- and isoflurane-induced neurotoxicity is associated with neuronal ferroptosis Zhang et al. (2022a)	Bupivacaine, lidocaine, propofol, ketamine and sevoflurane, *etc.* induce apoptosis and exhibit anticancer activity Mirshahidi et al. (2021); Tan et al. (2021)	Ketamine-associated cystitis is associated with autophagy Li et al. (2021a)	Potential toxicity of bupivacaine to intervertebral disc cells is associated with necroptosis Cai et al. (2018)	Prolonged high-dose propofol, sevoflurane and ketamine treatment induce cells pyroptosis Sun et al. (2019b)
Inhibits cell death under the influence of anaesthetics	DEX, propofol, and lidocaine, among others, inhibit ferroptosis in the IRI model Liu et al. (2021b); Lv et al. (2021); Lin et al. (2022); Tao et al. (2022); Wang et al. (2020a)	DEX, propofol, isoflurane, *etc.* inhibit apoptosis and protect important organs Lv and Li (2020)	Propofol sevoflurane, and DEX attenuate myocardial injury by modulating autophagy Jing et al. (2021)	Propofol, DEX, ketamine, and sevoflurane post-treatment attenuates necroptosis in inflammatory model cells Yin et al. (2020)	DEX, propofol, lidocaine and remimazolam *etc.* inhibit pyroptosis in inflammatory models Liu et al. (2020)

Most anaesthetics, in addition to anaesthesia, also have anti-tumour effects, such as bupivacaine, lidocaine and propofol, which can promote cell apoptosis and inhibit cancer cell proliferation (Mirshahidi et al., 2021; Tan et al., 2021). At clinically relevant concentrations, most anaesthetics are safe and have significance in protecting important organs. For example, clinical doses of propofol can block the pyroptosis of alveolar macrophages in renal ischemia/reperfusion (rI/R) rats and improve acute lung injury (ALI) caused by rI/R (Liu et al., 2020). However, long-term ketamine-induced bladder injury is usually associated with autophagy, and ketamine can upregulate autophagy-related proteins in bladder smooth muscle tissue and alter bladder angiogenesis (Li et al., 2021a). For oxidative stress, dexmedetomidine (DEX) can alleviate H_2_O_2_-induced oxidative stress and necroptosis, thereby protecting the body from damage (Yin et al., 2020). The discovery of ferroptosis broadened the exploration of anaesthetics for RCD. In a sepsis model, DEX can reduce ferroptosis and protect important organ damage by enhancing glutathione peroxidase 4 (GPX4) expression (Wang et al., 2020a). Etomidate inhibits ischaemia/reperfusion (I/R)-induced ferroptosis through the Nrf2 pathway and alleviates myocardial injury, which may supply a fresh idea for clinical reperfusion therapy (Lv et al., 2021). For cancerous cells, anaesthetics such as bupivacaine, propofol, and ketamine can induce ferroptosis and play an anticancer effect (He et al., 2021; Hao et al., 2022; Liu et al., 2021a). In an ALI model, both sevoflurane and DEX inhibited cellular ferroptosis and protected lung function (Liu et al., 2021b; Lin et al., 2022). However, ferroptosis is also involved in inhaled anaesthetic-induced neurotoxicity, and antiferroptosis treatment may provide new preventive ideas for perioperative anaesthesia complications (Zhang et al., 2022a).

**FIGURE 1:**
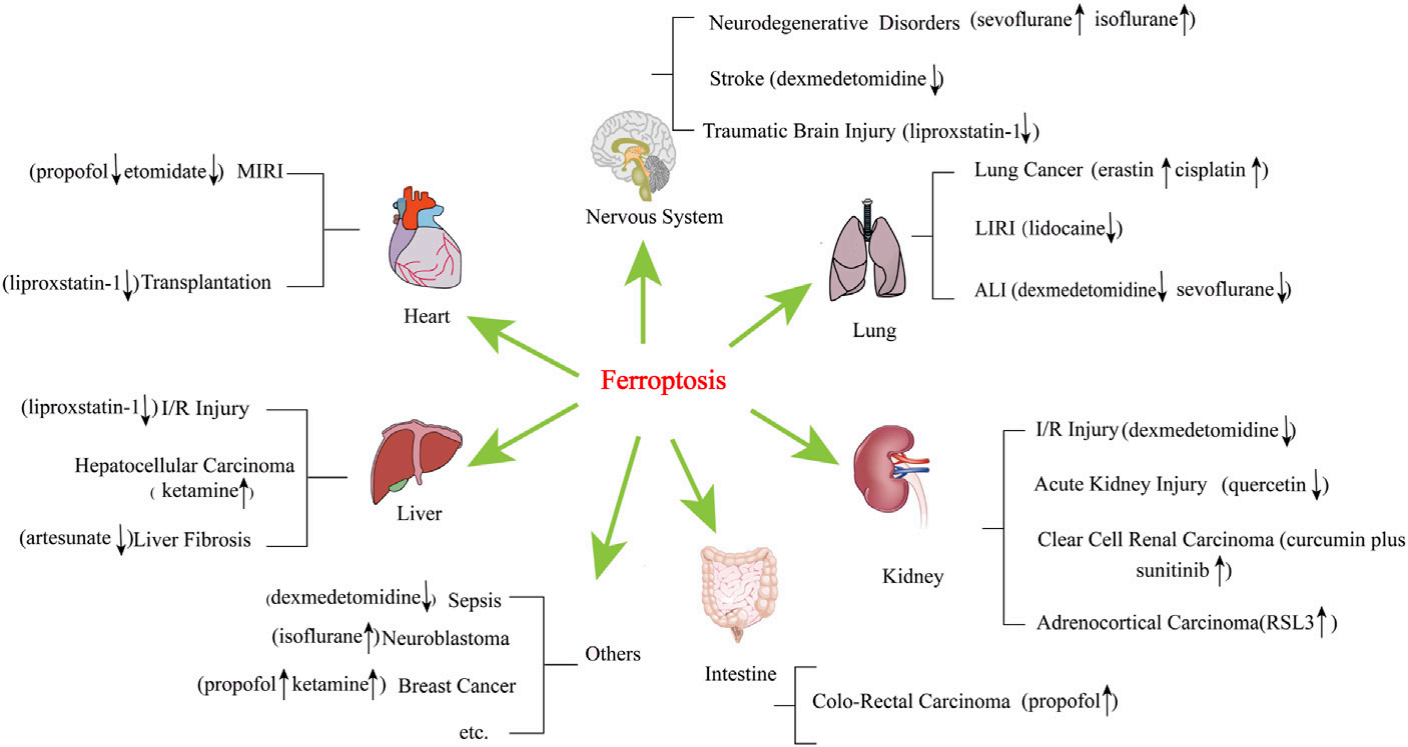
Diseases and narcotics are linked to ferroptosis. These diseases include acute lung injury, acute kidney injury, cancer, cardiovascular disease, neurodegenerative disease, and liver disease, among others. MIRI, myocardial ischaemia reperfusion injury; LIRI, lung ischaemia reperfusion injury; ALI, acute lung injury. “↑” represents promotion, “↓” represents inhibition.

Ferroptosis is regulated by various cellular metabolic pathways, including redox homeostasis (Stockwell et al., 2017), iron metabolism (Li et al., 2017), mitochondrial activity (Gao et al., 2019), and the metabolism of amino acids, lipids, and sugars (Yao et al., 2021), and is involved in the pathological mechanisms of various disease signalling pathways (Chen et al., 2020a; Tian et al., 2021; Yang et al., 2014). Ferroptosis plays an essential role in cancer, kidney injury, lung injury, nervous system disease, and ischaemia-reperfusion injury (IRI) (Chen et al., 2021a; Han et al., 2020) (Figure 1).

**FIGURE 2:**
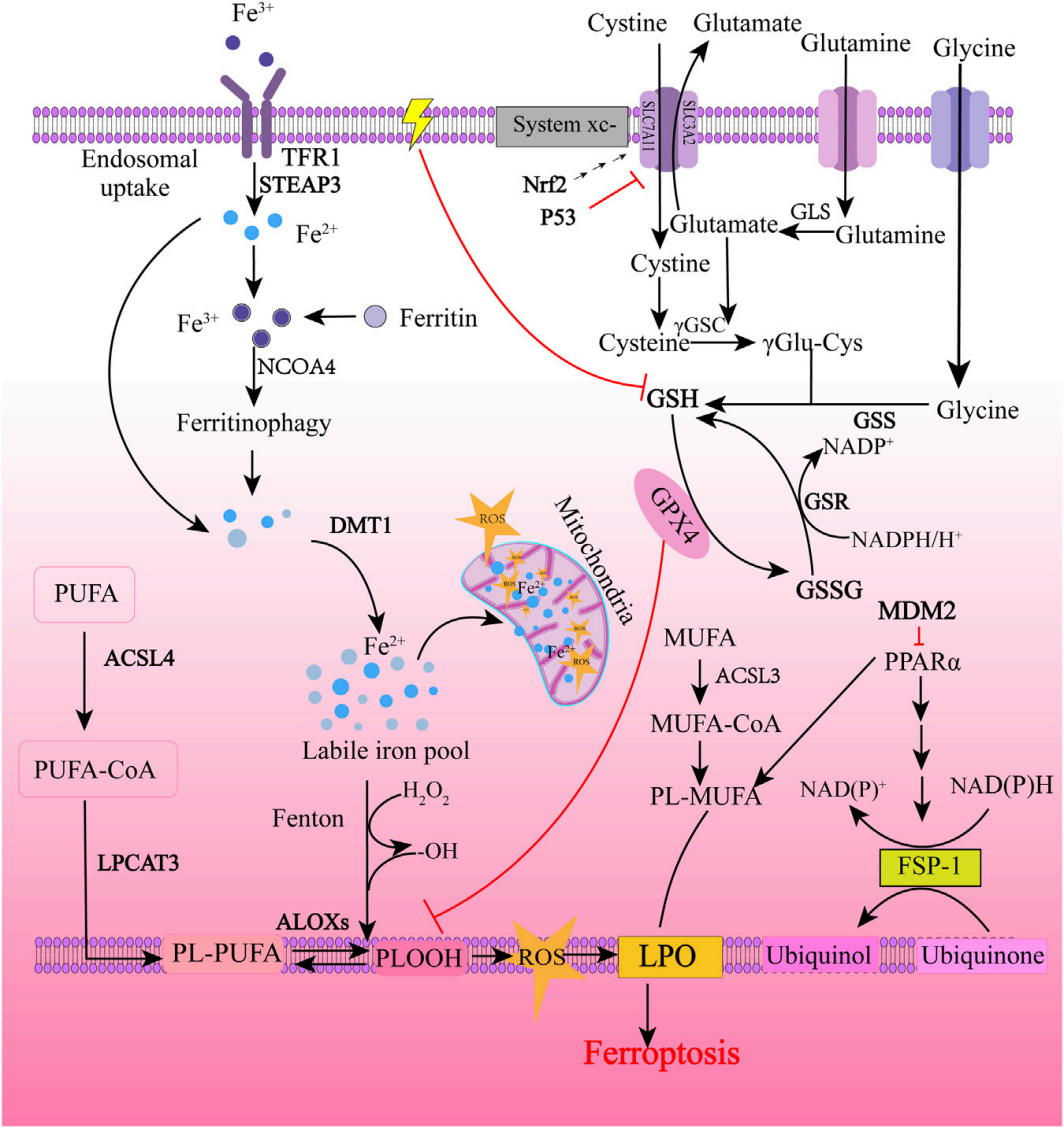
Molecular regulatory mechanism of ferroptosis. Ferroptosis is primarily initiated through two pathways: exogenous (transferrin dependent) pathways and endogenous (enzymatically regulated) pathways. The extrinsic pathway is initiated by cell membrane transporters such as cystine/glutamate transporter or by ferroportin, transferrin and lactoferrin activation. The endogenous pathway is activated by blocking intracellular antioxidant enzymes such as GPX4. The background color is from light to dark, with the light color representing exogenous pathways and the dark color representing endogenous pathways. TFR1, transferrin receptor 1; STEAP3, the six-transmembrane epithelial antigen of the prostate 3; NCOA4, nuclear receptor coactivator 4; DMT1, divalent metal transporter 1; ROS, reactive oxygen species; ACSL4, acyl-CoA synthetase long-chain family member 4; LPCAT3, lysophosphatidylcholine acyltransferase 3; ALOXs, arachidonic acid lipoxygenases; PUFA, polyunsaturated fatty acids; PUFA-CoA, polyunsaturated fatty acyl CoA; PL-PUFA, phospholipid-bound polyunsaturated fatty acids; PLOOH, phospholipid hydroperoxides; LPO, lipid peroxidation; Nrf2, nuclear factor erythroid 2-related factor 2; GLS, Glutaminase; γ-GSC, γ-glutamyl cysteinyl synthetase; γ Glu-Cys, γ glutamyl-cysteine; GSH, glutathione; GSS, glutathione synthetase; GSR, glutathione-disulfide reductase; GSSG, glutathione oxidized; GPX4, glutathione peroxidase 4; ACSL3, acyl-CoA synthetase long-chain family member 3; MUFA, monounsaturated fatty acids; MUFA-CoA, monounsaturated fatty acyl CoA; PL-MUFA, phospholipid-bound monounsaturated fatty acids; PPARα, peroxisome proliferator-activated receptor α; NADPH, nicotinamide adenine dinucleotide phosphate; NADP^+^, the oxidized form of NADPH; FSP-1, ferroptosis-suppressor-protein 1. “⊥” represents inhibition.

As shown in Figure 2, the regulatory mechanism of ferroptosis is mainly related to the regulation of iron metabolism, cystine-glutamate transporter (System xc-), glutathione (GSH), and GPX4 regulation, and lipid metabolism. I/R, iron overload, ionizing radiation, cytokines (such as INF-γ), certain drugs (for example, sulfasalazine, artemisinin, statins), and experimental reagents (such as erastin) can induce cell ferroptosis (Du et al., 2022; Su et al., 2020; Tang and Kroemer, 2020). Iron chelators, such as deferiprone (DFP), deferoxamine (DFO), ciclopirox (CPX), and 2,2-bipyridine (2,2-BP) (Dixon et al., 2012; Masaldan et al., 2018), and antioxidants, such as vitamin E, ferrostatin-1 (Fer-1), and liproxstatin-1 (Lip-1), showed inhibitory effects on ferroptosis (Kajarabille and Latunde-Dada, 2019).

In recent years, with the continuous development of anaesthesiology, the demand for anaesthesia in various clinical departments has been increasing. Anaesthesia is not limited to providing good surgical conditions and painlessness but also pays more attention to the regulation and maintenance of patients’ vital functions, such as stress response control. Its scope of work extends from the operating room to the ward, outpatient clinic, emergency room and other places. For anaesthesiologists, it is of great significance to improve anaesthesia management to reduce the burden of disease. The way anaesthesia is administered and the type of anaesthetic drug chosen in the hours during anaesthesia management may affect cancer recurrence months or years later (Sessler and Riedel, 2019). Anaesthetics can be divided into general anaesthetics and local anaesthetics. General anaesthetics are divided into inhalation anaesthetics and intravenous anaesthetics according to the route of administration. Commonly used inhalation anaesthetics include sevoflurane (Sev), isoflurane, and desflurane. Intravenous anaesthetics include sedatives (propofol, etomidate, *etc.*), analgesics (fentanyl, sufentanil, remifentanil, *etc.*). Commonly used local anaesthetics include lidocaine, bupivacaine, and ropivacaine. They all have different dosages and indications (Miller et al., 2005) (Table 2). Here, we review the effects of various anaesthetic drugs on ferroptosis-related molecular mechanisms and signalling pathways, aiming to provide a theoretical basis for targeting ferroptosis to prevent perioperative complications and improve postoperative outcomes for patients.

**TABLE 2:** Dosages, indications and potential effects on ferroptosis of some commonly used anaesthetics in the perioperative period.

Medicine	Way	Dosage	Indications	Potential effects on ferroptosis
Intravenous anaesthetics				
Propofol	intravenous	2–3 mg/kg	anaesthesia induction	Promotes ferroptosis in cancer cells Sun et al. (2022), inhibits ferroptosis in IRI cells Li et al. (2022)
	continuous intravenous injection	4–12 mg/kg.h	anaesthesia maintenance (adjusted according to compound drugs and conditions)	
Etomidate	intravenous	0.3–0.6 mg/kg	anaesthesia induction	Inhibits ferroptosis in IRI cells Lv et al. (2021)
Ketamine	muscle	3–5 mg/kg	sedation	Promotes ferroptosis in cancer cells He et al. (2021)
	muscle	5–7 mg/kg	anaesthesia	
	intravenous	0.5–1 mg/kg	sedation	
	intravenous	1–2 mg/kg	anaesthesia	
Dexmedetomidine	continuous intravenous injection	Load:0.3–1.0 µg/kg	anaesthesia maintenance	Inhibits ferroptosis in inflammatory diseases (such as ALI Lin et al.,(2022), sepsis She et al. (2021b), IRI Qiu et al., (2020))
		Maintenance:0.2–0.7 µg/kg.h	sedation	
Inhalation anaesthetics				
Sevoflurane	respiratory tract	MAC:1.85 vol%	anaesthesia maintenance	Promotes neuronal ferroptosis (in a time- and dose-dependent manner) Cheng et al. (2021)
Isoflurane	respiratory tract	MAC:1.15 vol%	anaesthesia maintenance	Promotes neuronal ferroptosis (in a time- and dose-dependent manner) Liu et al. (2021c)
Local anaesthetic				
	maximum dose (mg/kg)			
	No epinephrine		With 1:200,000 epinephrine	
Lidocaine	5		7	
Bupivacaine	2.5		3	
Levobupivacaine	2.5		3	
Potential effects on ferroptosis	Promotes ferroptosis in cancer cells Hao et al. (2022); Mao et al. (2021); Sun et al. (2021)			

## Mechanism of ferroptosis

### The classic system xc-/GSH/GPX4 pathway

The System xc-/GSH/GPX4 signalling pathway is the most important signalling pathway related to ferroptosis and plays an important role in intracellular lipid peroxidation. System xc- is a heterodimer consisting of the xCT light chain (encoded by the solute carrier family 7 members 11 gene; SLC7A11) and the 4F2 heavy chain (encoded by the SLC3A2 gene) (Sato et al., 1999). SLC3A2 is a single transmembrane protein that maintains the stability and proper membrane localization of the SLC7A11 protein (Nakamura et al., 1999). The 4F2 heavy chain is a ubiquitously expressed cell surface component shared with several other amino acid transport systems (Kandasamy et al., 2018). The xCT light chain consists of 12 specific transmembrane domains linked to the 4F2 heavy chain by extracellular disulfide bonds, selective for cystine and glutamate. System xc- exchanges intracellular glutamate with extracellular cystine at a 1:1 ratio (Bridges et al., 2012). Cystine in cells can be further converted into cysteine, and then glutathione (GSH) is synthesized, so System xc- plays a key role in maintaining intracellular GSH levels. The classic ferroptosis inducer erastin induces ferroptosis by inhibiting the activity of System xc-(26).

GPX4 is a key enzyme and a unique member of the mammalian selenium-dependent GPX family, which can degrade small molecule peroxides and certain lipid peroxides, inhibit lipid peroxidation, maintain lipid oxidation balance, and effectively repair lipid peroxidative damage (Brigelius-Flohé and Flohé, 2020). GSH is required as a substrate during ferroptosis, simultaneously converting reduced GSH to oxidized GSH (glutathione oxidized; GSSG) and converting toxic lipid hydroperoxides into nontoxic lipid alcohols, thereby reducing oxidative stress damage without affecting normal levels of cysteine or glutathione (Seibt et al., 2019), which is a negative regulator of ferroptosis. With reduced NADPH acting as the electron donor, GSH can be regenerated by reducing GSSG using glutathione reductase (GSR). Studies have shown that the regulation of cytosolic NADPH levels through metazoan spot homologue 1 (MESH1) can regulate ferroptosis (Ding et al., 2020). Therefore, GSH is considered to be an important factor in maintaining GPX4 activity. Moreover, RSL3 can irreversibly target selenocysteine binding to the active site of GPX4, thereby inhibiting the activity of GPX4 and inducing ferroptosis (Liang et al., 2019).

### p53-mediated ferroptosis

**FIGURE 3:**
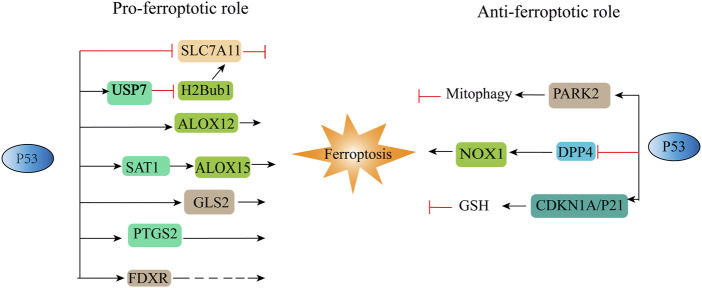
Positive and negative regulation of P53 to ferroptosis. ALOX12, arachidonic acid 12 lipoxygenases; ALOX15, arachidonic acid 15 lipoxygenases; CDKN1A/P21, cyclin-dependent kinase inhibitor; DPP4, dipeptidyl peptidase-4; FDXR, ferredoxin reductase; GLS2, glutaminases; PARK2, Parkinson disease 2; PTGS2, prostaglandin-endoperoxide synthase 2; SAT1, spermidine/spermine N1-acetyltransferase. “⊥” represents inhibition, “→“represents promotion.

p53 is a tumour suppressor gene that is generally associated with cell cycle arrest, apoptosis, and senescence (Brady et al., 2011; Li et al., 2012). Jiang et al. (2015) identified the relationship between p53 and ferroptosis for the first time, and the results showed that p53 can sensitize cells to ferroptosis. p53 not only inhibits the transcription of SLC7A11 through the p53 response element in the 5′ flanking region (Jiang et al., 2015) but also downregulates the expression of SLC7A11 by regulating USP7 and H2Bub1 (Wang et al., 2019). P53 also inhibits the cellular response to cystine uptake, resulting in decreased glutathione peroxidase activity, reduced cellular antioxidant capacity, and enhanced cellular susceptibility to ferroptosis (Guan et al., 2020). Nevertheless, activation of DNA damage by p53 has a dual role in promoting or inhibiting ferroptosis, depending on its gene target or binding protein (Kang et al., 2019; Zhang et al., 2022b) (Figure 3). Some metabolism-related genes, such as GLS2 (Gao et al., 2015), SAT1 (Ou et al., 2016), FDXR (Zhang et al., 2017) and ALOX12 (Chu et al., 2019), can act as p53 targets in different situations to promote ferroptosis. At the same time, p53 can also regulate CDKN1A, DPP4 and PARK2 to inhibit ferroptosis (Kang et al., 2019). Interestingly, p53 3KR (K117R, K161R, and K162R) acetylation-deficient mutants cannot induce cell cycle arrest, apoptosis, and senescence, but fully retain the ability to induce ferroptosis (Jiang et al., 2015). Another acetylation-deficient mutant, p53 4KR (KR98), failed to inhibit SLC7A11 expression or induce ferroptosis (Wang et al., 2016). Mechanistic analysis revealed that the combined removal of K117/161/162 and K98 acetylation blocked p53-mediated transcriptional regulation of TIGAR and GLS2, which is closely related to ferroptosis (Wang et al., 2016).

### Iron metabolism and ferroptosis

Iron is one of the essential nutrients for organisms and plays an important role in life. Iron in food is mainly Fe^3+^, which is absorbed by intestinal epithelial cells (Das et al., 2020), bound to transferrin (TF), transported through transferrin receptor 1 (TFR1) on the cell membrane surface, and enters cells by endocytosis (Cheng et al., 2004). In an acidic environment, Fe^3+^ is released from the conjugate, reduced to Fe^2+^ by the six transmembrane epithelial antigens of prostate 3 (STEAP3), and translocated into the cytoplasm by divalent metal transporter 1 (DMT1) (Torti and Torti, 2013). A small amount of Fe^2+^ forms an unstable iron pool (LIP) (Zanninelli et al., 2002), excess iron is mainly stored in ferritin in a redox-inactive form, and part of Fe^2+^ is transported out of the cell by the action of ferroportin 1 (FPN1). Changes in any of these steps may have an impact on iron metabolism. In general, the balance of intracellular iron is reflected in the balance between iron input, storage, and output (Ganz, 2013). Too much iron is harmful. A certain amount of free iron is necessary for ferroptosis to occur. On the one hand, iron can increase the activity of metabolic enzymes such as arachidonic acid lipoxygenase (ALOX) and promote ferroptosis by stimulating the production of lipid ROS (Chen et al., 2020b). On the other hand, iron is able to act as a ferrous ion and react with H_2_O_2_ to generate hydroxyl radicals OH and ROS in the Fenton reaction (Toyokuni, 2002), leading to cell death through peroxide damage on DNA, protein and membrane lipids. In conclusion, abnormal iron metabolism induces ferroptosis, which is a necessary pathway for ferroptosis.

### Lipid metabolism and ferroptosis

Lipid metabolome genes have shown that during ferroptosis, polyunsaturated fatty acids (PUFAs) such as epinephrine (ADA) and arachidonic acid (AA), are most susceptible to peroxidation, damaging lipid bilayers and affecting cell membrane function (Kagan et al., 2017). PUFAs contain easily extractable bisallyl hydrogen atoms and are susceptible to lipid peroxidation (Yang et al., 2016). Acyl-CoA synthetase long-chain family member 4 (ACSL4) belongs to the long-chain acyl-CoA synthetase family and is a crucial enzyme controlling fatty acid metabolism (Kuwata and Hara, 2019). ACSL4 and lysophosphatidylcholine acyltransferase 3 (LPCAT3) promote the incorporation of PUFAs into phospholipids to form phospholipid-bound polyunsaturated fatty acids (PL-PUFA). PL-PUFA is prone to ALOX-induced lipid peroxidation, which is oxidized by LOXs or POR to form lipid peroxides, which ultimately leads to lipid bilayer disruption, thereby promoting ferroptosis by affecting cell membrane function (Du et al., 2022). ACSL4 is considered a key regulator of ferroptosis (Kagan et al., 2017; Yuan et al., 2016). The key enzymes in the synthesis of the unsaturated phospholipids ACSL4 and LPCAT3 are associated with RSL3-induced ferroptosis (Hadian and Stockwell, 2020). At the same time, PUFAs are competitively affected by monounsaturated fatty acids (MUFAs), and MUFAs do not have bisallyl sites, so they are not prone to peroxidation, which means that exogenous MUFAs may produce ferroptosis resistance. While MUFA-induced ferroptosis is dependent on ACSL3, ACSL3 converts MUFAs to acyl-CoA esters that bind to membrane phospholipids and reduce the sensitivity of plasma membrane lipids to lethal oxidation, thereby protecting cancer cells from ferroptosis attack (Magtanong et al., 2019).

### Other ferroptosis regulatory pathways

Other major regulatory mechanisms of ferroptosis include the voltage-dependent anion channels (VDACs) pathway and the ferroptosis suppressor protein 1 (FSP1)/coenzyme Q10 (CoQ10) signalling pathway. The FSP1/CoQ10 signalling pathway is a new pathway independent of the System xc-/GSH/GPX4 signalling pathway discovered in recent years (Bersuker et al., 2019). FSP1 is an oxidoreductase, which can reduce CoQ10 (also known as ubiquinone) to reduced CoQ10 (also known as ubiquinol) under the action of NADPH, capture lipid peroxy radicals that mediate lipid peroxidation, and inhibit lipid peroxidation. Propagation of oxides inhibits ferroptosis (Doll et al., 2019). VDACs are a class of porin ion channels located in the outer mitochondrial membrane that mainly transport ions and metabolites between the cytoplasm and mitochondria (Kim et al., 2019; Shoshan-Barmatz et al., 2010). Many researchers have found that erastin can act on VDACs to keep them open, thereby enhancing mitochondrial respiration and increasing ROS production and promoting ferroptosis (Yagoda et al., 2007; Yang et al., 2020; Zhao et al., 2020). Interestingly, Yagoda et al. (2007) found that downregulation of VDAC2 and VDAC3 protein levels can reduce mitochondrial ROS production and lead to the development of tolerance to erastin-induced ferroptosis. Mitochondria are the regulatory centres of cellular metabolism and energy metabolism and are constantly remodelled through fission and fusion. Mitochondria are also involved in cellular functions such as ATP production, intracellular calcium regulation, and ROS production (Roy et al., 2019). All of the above evidence demonstrates the indispensable role of mitochondria in the regulation of ferroptosis.

## The effect of anaesthetics on ferroptosis

### The effects of intravenous anaesthetics on ferroptosis

Propofol, etomidate, and ketamine are commonly used intravenous anaesthetics in clinical practice. Among them, propofol is an alkylphenol compound due to its fast induction, rapid recovery, and lack of easy accumulation after continuous infusion. Propofol also has specific nonanaesthetic effects, such as antiemetic, anti-inflammatory, antioxidant, antitumour, neuroprotective, and antiplatelet aggregation effects (Bateman and Kesselheim, 2015; Jiang et al., 2018). As one of the most commonly used intravenous anaesthetics, propofol is widely applied for anaesthesia induction and intraoperative maintenance. Similar to the endogenous antioxidant vitamin E, the molecular structure of propofol contains phenolic hydroxyl groups, so it can directly scavenge ROS and inhibit lipid peroxidation (Wilson and Gelb, 2002). At the same time, propofol can inhibit inflammation (Wu et al., 2018), reduce oxidative stress and ameliorate IRI (85), but I/R can induce ferroptosis. In a mouse cardiac IRI model, treatment with Fer-1 effectively reduced ferroptosis-induced myocardial infarction, improved left ventricular contractility, and reduced left ventricular remodelling (Li et al., 2019a). In intestinal I/R, ACSL4 is critical in this consistent process, and Fer-1 attenuates damage caused by intestinal ischaemia (Li et al., 2019b). These all suggest that ferroptosis is related to I/R. Studies have shown that propofol can increase the levels of ferritin heavy chain-1 (FTH1), xCT, or GPX4, improve the antioxidant capacity of cardiomyocytes *via* iron level reduction and inhibit cell ferroptosis through the AKT/P53 signalling pathway (Li et al., 2022). AKT is a serine/threonine-specific protein kinase essential for regulating the response to myocardial I/R injury (Yin et al., 2013). Phosphorylation of AKT can promote the binding of murine double minute 2 (MDM2) to p53, leading to p53 degradation (Wang et al., 2020b) and protecting myocardial cells from myocardial ischaemia‒reperfusion-induced ferroptosis. Therefore, propofol can protect some cells from ferroptosis. Propofol also protects neurons by reducing ferroptosis, as propofol (50 μM) reduces erastin-induced high iron concentrations, lipid peroxides, and excess reactive oxygen species (Xuan et al., 2022). However, animal experiments and *in vitro* cell experiments consistently show that propofol has the potential to inhibit the malignancy of primary cancers (Sessler and Riedel, 2019), promoting ferroptosis in cancer cells. Clinically relevant concentrations (5 µg/ml, 10 µg/ml, 20 µg/ml) of propofol can inhibit the viability of cervical cancer cells *in vitro* and enhance the morphological changes related to ferroptosis and mitochondrial atrophy in HeLa cells induced by paclitaxel (Zhao et al., 2022). It is manifests as increased intracellular Fe^2+^ concentrations, inhibition of the SLC7A11/GPX4 and ubiquinol/FSP1/CoQ10 pathways, upregulation of the expression of the ferroptosis regulators ACSL4, TFRC, *etc.*, to promote ferroptosis in C-33A and HeLa cells. However, another *in vitro* study showed that pretreatment with 10 µg/ml propofol could induce ferroptosis and inhibit the proliferation of MDA-MB-231 breast cancer cells. Propofol induced ferroptosis through the P53-SCL7A11-GPX4 pathway but not the ubiquinone-FSP1-ubiquinol axis, even though propofol downregulates FSP1 expression (Sun et al., 2022). These results are based on *in vitro* experiments and require further verification in animal models. In addition, unlike directly administering propofol in *in vitro* cell experiments, the common preparation in clinical practice is a propofol emulsion, which may lead to different antitumour effects and needs to be further explored. Propofol at clinically relevant concentrations promotes ferroptosis in cancer cells, which is inconsistent with previous claims and exhibits antiferroptotic effects in ischemia-reperfusion injury disease. The possible reason for this contradiction is that propofol acts on different disease models and the final response results are different. What controls the ferroptosis signaling pathway network to show different outcomes in different diseases are still unclear, which needs to be further answered in the future. Studies have shown that different propofol concentrations may have both cytoprotective and cytotoxic effects with prolonged use (Lin et al., 2015). However, whether high concentrations and prolonged use of propofol lead to ferroptosis, especially exposure duration, still needs further study.

Etomidate is also a commonly used intravenous anaesthetic, with few side effects on blood circulation, and rapid recovery. Etomidate can reduce the expression of myocardial fibrosis-related proteins (α-smooth muscle actin and collagen II) and the secretion of inflammatory factors (IL-6, IL-1β, and TNF-α) in myocardial ischaemia reperfusion injury (MIRI) rats, reducing superoxide dismutase content, glutathione activity, and GPX4 expression while upregulating malondialdehyde, iron, acyl-CoA and synthase long-chain family member 4 levels and alleviating I/R-induced cardiac ferroptosis (Lv et al., 2021). Experimental studies have shown that the effects of etomidate on I/R-induced myocardial fibrosis and inflammation can be reversed by erastin (Lv et al., 2021), a well-established inducer of ferroptosis (DeHart et al., 2018). It can be seen that etomidate has both anti-inflammatory and anti-ferroptosis effects on the IRI model. The relationship between ferroptosis and inflammation is intricate, and the specific mechanism still needs to be further explored.

Ketamine is a fast-acting anaesthetic with a short half-life and mild respiratory depression. Ketamine may lead to abuse; otherwise, it is an ideal anaesthetic. In addition to anaesthetic effects, ketamine also showed anti-inflammatory, anti-anxiety, anti-depressant, analgesic, and other effects (Roytblat et al., 1998; Wolff and Winstock, 2006; Zarate et al., 2006) and potential antitumour properties. Ketamine was applied to treat breast cancer, and the results showed that ketamine could increase lipid ROS, malondialdehyde (MDA), and Fe^2+^ in breast cancer cells, inhibiting the expression of the ferroptosis factor GPX4 by attenuating KAT5 on the GPX4 promoter region and reducing histone H3 lysine 27 acetylation (H3K27ac) and the enrichment of RNA polymer II (RNA pol II), ultimately leading to breast cancer cell ferroptosis (Li et al., 2021b). In ketamine- or Sev-induced neurotoxicity, ketamine or Sev exposure caused upregulation of excitatory N-methyl-D aspartate receptor (NMDAR) subunits (Wu et al., 2020), which may be a compensatory mechanism (Haseneder et al., 2013; Liu et al., 2013; Sinner et al., 2015). In addition, both Sev and ketamine led to the upregulation of RASD1, which can form a ternary complex with DMT1 and PAP7, thereby enhancing the capability of NMDARs to activate DMT1 for iron uptake (Cheah et al., 2006; Chen et al., 2015a). DMT1-mediated iron influx promotes iron overload and stimulates lysosome iron release (White et al., 2016), causing iron-induced cellular damage. These results suggest that some non-narcotic effects of ketamine are closely related to ferroptosis.

### The effects of inhaled anaesthetics on ferroptosis

**FIGURE 4:**
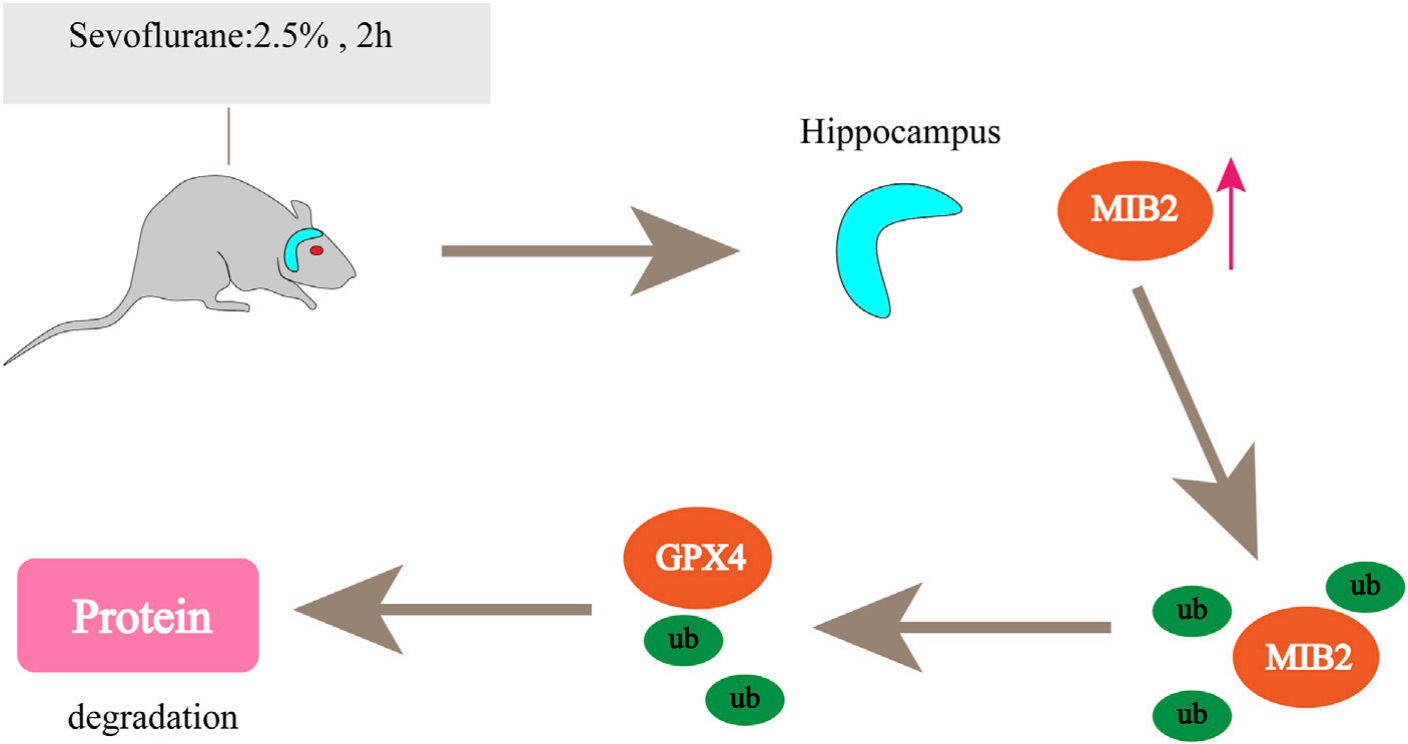
Sevoflurane increases MIB2 expression in the mouse hippocampus. MIB2 can directly bind to GPX4, regulate its stability and ubiquitination, and regulate ferroptosis in mouse hippocampal neurons. “↑” represents promotion.

Currently, ferroptosis-related inhalation anaesthetics mainly include Sev and isoflurane. There is growing evidence showing that inhaled anaesthetics induce neuronal cell death, especially in older patients, and increase the risk of postoperative cognitive dysfunction (POCD) (Hudson and Hemmings, 2011). Iron is essential for neural development, neurotransmitter synthesis, and mitochondrial function (Singh et al., 2014). Excess iron can lead to neurotoxicity and cognitive impairment. In neurons, ferroptosis was induced by Sev through iron regulatory protein (IRP2)- and transferrin (TFR1)-mediated iron overload (Wu et al., 2020). In Sev-exposed neurons, anaesthesia increased MIB2 expression in mouse hippocampal neurons and tissues; MIB2 knockout attenuated Sev-induced neuronal ferroptosis, while GPX4 knockout reversed this effect (Zhao et al., 2021). MIB2 can directly bind to GPX4 and regulate its stability and ubiquitination, thus regulating ferroptosis in mouse hippocampal neurons (Figure 4). ACSL4 is also involved in Sev-induced neuronal ferroptosis. Sev-induced ferroptosis is inhibited by ACSL4 downregulation through the 5′ AMP-activated protein kinase (AMPK)/mammalian target of rapamycin (mTOR) signalling pathway (Cheng et al., 2021). Therefore, targeting ACSL4 may be a potential therapeutic strategy to alleviate Sev-induced POCD. Another study confirmed that Sev could regulate the development of various tumours and exert an antitumour effect on ovarian cancer (Zhang et al., 2019a), colorectal cancer (Fan et al., 2019), lung cancer (Liang et al., 2012), glioma (Xu et al., 2022), *etc.* Sev treatment also induced ferroptosis in glioma cells. Basic experiments have shown that Sev can inhibit cell viability in a dose-dependent manner in U87 and U251 glioma cells, increase ROS levels and Fe^2+^ concentrations, upregulate the expression of transferrin, ferritin, and Beclin-1, and induce glial tumour cell death. According to Sev transcriptional sequencing, the expression of ferroptosis- and mitophagy-related gene-activating transcription factor 4 (ATF4) was enhanced, and Sev induced ferroptosis in glioma cells by activating the ATF4-CHAC1 pathway (Xu et al., 2022). In lipopolysaccharide (LPS)-induced ALI, in BEAS-2B cells, Sev inhibited ferroptosis *via* haem oxygenase-1 (HO-1) upregulation (Liu et al., 2021b). HO-1, as the initiating and rate-limiting enzyme of haem catabolism, can catalyse the conversion of haem to biliverdin, carbon monoxide, and iron (Zhang et al., 2018a), so HO-1 may be a potential source of intracellular iron on which ferroptosis depends and is beneficial for cancer therapy (Adedoyin et al., 2018). However, in the study by LIU, the increased HO-1 after Sev treatment did not cause iron overload or GPX4 overexpression. This may be because HO-1 also has anti-ferroptosis properties (Su et al., 2019), which are particularly reflected in its protective effect against lung injury (Dong et al., 2020; Zampetaki et al., 2003). This result may be due to the different sensitivities of drugs in different organs and the different expression of related genes in different physiological states of the body, and further research is needed in the future.

Several animal and clinical studies have confirmed that isoflurane and other anaesthetics (including sevoflurane, midazolam, *etc.*) can cause neurotoxicity in the brain. However, the neurotoxicity caused by isoflurane is limited to the developing brain and has not been found in the adult brain (Andropoulos, 2018; McCann and Soriano, 2019). A study by Liu et al. (2021c) showed that isoflurane induces ferroptosis in hippocampal neurons in a time- and dose-dependent manner. Isoflurane downregulated SLC7A11 protein levels, resulting in cellular cystine, cysteine, and GSH depletion, which disrupted cellular redox homeostasis and ultimately led to ferroptosis. Meanwhile, isoflurane exposure also increased the activity of cytochrome c oxide/complex IV in the mitochondrial electron transport chain (ETC), further promoting cysteine deprivation-induced lipid ROS accumulation and ultimately resulting in ferroptosis. Isoflurane-induced ferroptosis could be reversed by a ferroptosis inhibitor (ferrostatin-1) and a mitochondrial protectant (dimethyl fumarate; DMF). Similar results were obtained in another basic study. Isoflurane upregulated the phosphorylation of Beclin1, which formed the Beclin1-SLC7A11 complex, affected the activity of the cystine/glutamate transporter, and regulated ferroptosis in SH-SY5Y neuroblastoma cells by inhibiting the release of glutamate and decreasing intracellular GSH activity (Liu et al., 2019). In conclusion, neuronal ferroptosis is closely related to neurotoxicity after inhalation general anaesthesia in elderly patients. We speculate that anti-ferroptosis preconditioning may improve postoperative learning and memory, and further clinical research is needed.

### The effects of local anaesthetics on ferroptosis

Lidocaine is a cationic lipophilic molecule with anaesthetic and antiarrhythmic properties. As a commonly used local anaesthetic in clinical practice, it exerts its effect by interacting with lipid membranes (Santa-Maria et al., 2019). An *in vitro* study found that the expression levels of FTH1 and GPX4 were significantly reduced by hypoxia/reoxygenation (H/R) in A549 cells. Administration of lidocaine reversed the expression levels of FTH1 and GPX4 to inhibit ferroptosis (Ma et al., 2022). Interestingly, 5 mM lidocaine appeared to have similar effects on ferroptosis markers and claudin as 10 mM lidocaine. However, 10 mM lidocaine had stronger effects on cell viability, the inflammatory response, and oxidative stress than 5 mM lidocaine. This may be due to the different susceptibilities of lidocaine to the biological activity of different cells. Lidocaine can promote ferroptosis in ovarian and breast cancer cells by enhancing the expression of microRNA-382-5p (miR-382-5p) in cells and downregulating SLC7A11 (123). However, it may just be one of the downstream mechanisms by which lidocaine inhibits cancer progression, so further research is needed. The effect of lidocaine on ferroptosis is not absolute. It not only exerts a ferroptotic effect on tumour cell lines but also exerts an antiferroptotic effect on I/R-induced cell damage. This may be because lidocaine has different responses based on the genomes of different cell lines, and it is necessary to delve deeper into its mechanism to find a common pathway. Furthermore, whether ferroptosis is a necessary regulatory pathway for lidocaine is still unclear, and more *in vivo* experiments are required for future investigation.

Bupivacaine, a commonly used clinical local anaesthetic with odium channel blocking properties, is administered through local infiltration and epidural and intrathecal anaesthesia. At the same time, bupivacaine can also inhibit the growth of tumour cells (Dan et al., 2018). In an *in vitro* study, the Fe^2+^ concentration in T24 and 5637 cells was upregulated by bupivacaine in a concentration-dependent manner. At the protein level, bupivacaine inhibited the expression of solute carrier family 7 members 11 (SLC7A11; also commonly known as xCT) and GPX4 while increasing ROS levels. Further results showed that bupivacaine decreased mitochondrial membrane potential, decreased GSH, increased MDA levels, and attenuated the phosphorylation of PI3K, Akt, and mTOR (Hao et al., 2022). These data suggest that bupivacaine promotes ferroptosis in bladder cancer cells.

Levobupivacaine (L-bupivacaine), a new long-acting amide local anaesthetic, is an isomer of bupivacaine and has potential antitumour properties. Over the past few decades, local anaesthetics such as levobupivacaine, bupivacaine, and lidocaine have been shown to affect the progression of a variety of cancers, including gastric, breast, and lung cancers (Dan et al., 2018; Li et al., 2018). Levobupivacaine can inhibit the proliferation, invasion and migration of non-small cell lung cancer (NSCLC) cells and induce their apoptosis (Meng et al., 2021). Meanwhile, levobupivacaine treatment enhanced erastin-induced growth inhibition of NSCLC cells, significantly increased the levels of ROS, iron and Fe^2+^ in NSCLC cells, and promoted tumour cell ferroptosis. Above studies suggest that the antitumor properties of local anaesthetics are associated with ferroptosis. However, such research is still very lacking and needs further studies.

### The effects of anaesthetic adjuvants on ferroptosis

As a novel, highly selective, and particular α2 adrenergic receptor (α2-AR) agonist with sedative, analgesic, anxiolytic and hypnotic effects, DEX is widely used in intensive care and anaesthesia adjuvants (Sun et al., 2019a). *In vivo* studies showed that intraperitoneal injection of DEX 20 µg/kg 1 h before surgery alleviated LPS-induced ALI in mice and reversed LPS-induced upregulation of MDA and downregulation of GPX4 in the lung. It has been suggested that DEX can protect ALI well through ferroptosis (Lin et al., 2022). Another study reached similar conclusions; She et al. (2021a) observed the mitochondrial structure of pulmonary veins in septic rats induced by caecal ligation and puncture (CLP) with transmission electron microscopy. The results showed that mitochondrial fission was excessive, and the morphology was fragmented, which seriously affected mitochondrial function. However, DEX treatment significantly improved mitochondrial morphology and significantly reduced the frequency of mitochondrial fission. DEX inhibited mitochondrial fission by regulating the translocation of dynamin-related protein 1 (Drp1) while reducing ER-MITO contacts in vascular endothelial cells (VECs) through actin polymerization blockade (She et al., 2021a). Hierarchical clustering heatmaps and radar maps were applied to analyse the influence of DEX administration. The results showed that γ-glutamylcysteine, D-ribose 5-phosphate, L-malate and L-glutamate generation were significantly promoted, suggesting metabolic reprogramming from glycolysis to the pentose phosphate pathway (PPP). Ferroptosis was inhibited by DEX through the PPP metabolic, G6PD/Nrf2 (nuclear factor erythroid 2-related factor 2), GSH, free radical scavenging, and lipid peroxidation pathways (She et al., 2021b). Nrf2 is a redox-sensitive transcription factor. When prooxidant levels are elevated, Nrf2 can no longer be sequestered by Keap-1 and subsequently translocates into the nucleus, where it binds to promoters and activates the transcription of target genes. At present, the mechanism by which DEX activates Nrf2 is still unclear. Xu et al. (2021) reported that Nrf2 could bind to α2-AR so DEX-induced α2-AR activation may be responsible for the upregulation of Nrf2. However, the detailed mechanism requires further investigation. Subsequent members of this group (Wang et al., 2020a) further found that CLP surgery-induced cardiomyocyte injury in sepsis was alleviated by DEX through regulation of HO-1 iron concentration and GPX4. In ischaemia-reperfusion models, DEX can regulate the import and export of iron through the JNK/SP1 and STST4/SP1 signalling pathways, prevent iron efflux by reducing FPN1 expression or by reducing its influx of TFR1 and DMT1, maintain iron homeostasis and protect cells from ferroptosis (Qiu et al., 2020). Meanwhile, DEX can also alleviate ferroptosis-mediated renal ischemia/reperfusion injury and inflammation through α2-AR inhibition of ACSL4 signalling (Tao et al., 2022). In general, DEX, as a commonly used adjuvant drug for anaesthesia, has anti-ferroptosis effect on inflammatory diseases and has protective significance for the body. However, more research is needed to focus on the regulatory mechanism of DEX in inflammatory diseases *via* ferroptosis.

### Narcotics and ferroptosis-related noncoding RNA

MicroRNAs (miRNAs) are defined as endogenous short noncoding RNAs. miRNAs typically function by interacting with mRNAs of target genes and hindering the successful translation of their proteins, and miRNA regulation can occur in various biological processes during tumour development, such as growth, angiogenesis, and metastasis (Lee and Dutta, 2009). Wang et al. (He et al., 2021) proposed that ketamine-induced ferroptosis in hepatoma cells *in vivo* or *in vitro* was related to the miRNA profile, which was manifested as inhibiting GPX4 expression by reducing lncRNA PVT1 and promoting ferroptosis by upregulating miR-214-3p. A luciferase reporter assay revealed a direct interaction between miR-214-3p and the sponge of the GPX4 3′UTR, and lncPVT1 promoted GPX4 expression by sponging miR-214-3p. Currently, miR-214-3p is an inhibitory miRNA that has been extensively studied in cancer and has been proposed as a diagnostic and prognostic biomarker for several cancers (Fang et al., 2019a; Han et al., 2019; Lu et al., 2020). Propofol inhibited the expression of STAT3 by upregulating miR-125b-5p in gastric cancer cells, and STAT3 overexpression reversed the accumulation of Fe^2+^, ROS, MDA levels in erastin-treated SGC7901 and BGC823 cells by propofol, thereby demonstrating that propofol can enhance the ferroptosis of gastric cancer cells (Liu et al., 2021a). Additionally, in SGC7901 gastric cancer cells, levobupivacaine treatment elevated the level of miR-489-3p and further acted as a sponge for SLC7A11 to inhibit its expression, thereby promoting ferroptosis in gastric cancer cells (Mao et al., 2021). Transfer RNA‐derived small RNAs (tsRNAs) are an abundant type of noncoding small RNAs that play multiple roles in different physiological processes (Kim et al., 2017; Wang et al., 2020c). There is increasing evidence that tsRNA is capable of affecting gene expression at the posttranscriptional level by targeting the 3′UTR, showing a mechanism similar to that of miRNAs (Canella et al., 2010; Haussecker et al., 2010). Recent evidence has shown that DEX reverses LPS-induced ferroptosis in LPS-induced ALI. RNA sequencing was performed to explore aberrantly expressed tsRNAs between the LPS and LPS + DEX groups in lung tissue. The results showed that tsRNA-1018, tsRNA-3045b, tsRNA-5021a, and tsRNA-1020 were downregulated, and tsRNA-3025b was upregulated (Lin et al., 2022). Sev induces ferroptosis in a variety of diseases, including glioma. In addition to regulating the expression of mRNAs, Sev was also found to influence the expression of a few lncRNAs in glioma cells (Xu et al., 2022).

LncRNAs are noncoding RNAs over 200 nt in length that can interact with RNA, proteins, and DNA. The lncRNA TMEM161B-AS1 was discovered to promote certain malignant behaviours and temozolomide resistance by enhancing the expression of multiple ferroptosis-related genes (Chen et al., 2021b). However, the disadvantage is that although the above studies have shown that the effect of anaesthetics on ferroptosis is related to noncoding RNA, the related epigenetic regulation has not been discussed, and further exploration may be required in the future. Taken together, these findings contribute to further understanding of the mechanisms underlying the antitumour properties of anaesthetics. It is demonstrated that anaesthetics may act as tumour suppressors to inhibit cancer growth through ferroptosis-related noncoding RNAs and could be a new therapeutic strategy for cancer.

## Links between anaesthetic-related ferroptosis and diseases

Accumulating clinical data suggest that human disease is linked to ferroptosis-related modulators (activators or inhibitors) and pathways (Jiang et al., 2021; Tang et al., 2021). Therefore, targeting ferroptosis by pharmacological modulators may represent a possible approach to the treatment of multiple pathologies. In surgical operations, the selection and application of anaesthetics is likely to affect the development of the patient’s postoperative condition. Although the physiological function of ferroptosis remains unclear, its role in a large number of human diseases, such as cancer, neurodegeneration, IRI, and other pathological conditions, has been widely documented. As discussed in Table 3, the induction or inhibition of ferroptosis by anaesthesia drugs is associated with a variety of disease mechanisms, which further shows that the use of anaesthesiologists in each choice of narcotics is crucial Here, we will try to illustrate the role of ferroptosis in diseases through several good examples.

**TABLE 3:** Effects of narcotics on ferroptosis.

Treatment	Source cells	Disease	Expression	Molecular axis	Functions	References
Bupivacaine	T24 and 5637 cells	Bladder cancer	Up	PI3K/AKT	Restrains the expression of xCT and GPX4	Hao et al. (2022)
Levobupivacaine	HGC27 and SGC7901 cells	Gastric cancer	Up	miR-489-3p/SLC7A11	Represses SLC7A11 expression	Mao et al. (2021)
Lidocaine	SKOV-3 and T47D cells	Ovarian cancer and breast cancer	Up	MiR-382-5p/SLC7A11	Inhibits SLC7A11 expression	Sun et al. (2021)
	A549 cells	Lung ischaemia‒reperfusion injury	Down	p38 MAPK	Upregulates FTH1 and GPX4 expression levels	Ma et al. (2022)
Dexmedetomidine	VECs	Sepsis	Down	Nrf2/G6PD	Suppresses oxidative damage and modulate metabolic reprogramming	She et al. (2021b)
	Lung tissue cells	ALI	Down	PI3K/AKT, MAPK, NF-kappaB	tsRNA-mRNA	Lin et al. (2022)
	cardiomyocytes	Sepsis	Down	HO-1	Enhances GPX4 expression and reduces the expression of HO-1	Wang et al. (2020a)
	SK-N-SH cells	Cerebral ischaemia/reperfusion injury	Down	JNK/Sp1, STAT4/Sp1	Regulates iron importers and exporters	Qiu et al. (2020)
	HEK293T cells	Renal ischaemia/reperfusion injury	Down	ACSL4	Inhibits ACSL4 signalling	Tao et al. (2022)
Sevoflurane	U87, U251 cells	Glioma	Up	ATF4-CHAC1	Increases ATF4 expression and decreases the expression of GPX4	Xu et al. (2022)
	SH-SY5Y cells	POCD	Up	AMPK/mTOR	Increases the expression of ACSL4	Cheng et al. (2021)
	Rat hippocampal neuronal cells	POCD	Up	MIB2/GPX4	Increases MIB2 expression and reduces GPX4 protein expression	Zhao et al. (2021)
	BEAS-2B cells	ALI	Down	HO-1	Upregulates HO-1 expression	Liu et al. (2021b)
	Rat hippocampal neuronal cells	Neurodegenerative disease	Up	RASD1-DMT1	Upregulates NMDAR subunits	Wu et al. (2020)
Isoflurane	Rat hippocampal neuronal cells	Neurodegenerative disease	Up	System xc-	Decreases System xc- expression and increases the activity of cytochrome c oxidase/complex IV in mitochondrial ETC	Liu et al. (2021c)
	SH-SY5Y cells	Neurodegenerative disease	Up	System xc-	Upregulates Beclin 1 phosphorylation and inhibits System xc- activity	Liu et al. (2019)
Propofol	C-33A and HeLa cells	Cervical cancer	Up	SLC7A11/GPX4, ubiquinol/CoQ10/FSP1, YAP/ACSL4/TFRc	Increases intracellular Fe^2+^, decreases the expression of GPX4, SLC7A11, ubiquinol, CoQ10, FSP1 and upregulates the expression of ACSL4, TFRc	Zhao et al. (2022)
	MDA-MB-435 cells	Breast cancer	Up	P53/SLC7A11/GPX4	Inhibits GPX4 and SLC7A11 activity	Sun et al. (2022)
	H9C2 cells	Ischaemia/reperfusion injury	Down	AKT/P53	Promotes P53 degradation, improves the levels of FTH1, xCT or GPX4 and reduces iron levels	Li et al. (2022)
	SGC7901 and BGC823 cells	Gastric cancer	Up	MiR-125b-5p/STAT3	Inhibits the expression of GPX4 and SLC7A11, reduces GSH levels, enhances MDA levels	Liu et al. (2021a)
Etomidate	Cardiomyocytes	Myocardial ischaemia reperfusion injury	Down	Nrf2/HO-1	Upregulates glutathione activity and GPX4 expression, reduces the level of ACSL4	Lv et al. (2021)
Ketamine	Breast cancer cells	Breast cancer	Up	KAT5/GPX4	Inhibits the expression of GPX4	Li et al. (2021b)
	Liver cancer cells	Liver cancer	Up	LncRNA PVT1/miR-214-3p/GPX4	Inhibits the expression of GPX4	He et al. (2021)

### Cancer

Bupivacaine, levobupivacaine, lidocaine, sevoflurane, propofol and ketamine can induce ferroptosis in cancer cells and exert an antitumour effect. Different types of cancer cells have different susceptibilities to ferroptosis. Among the eight different tissue types used in the NCI (National Cancer Institute) Developmental Therapy Program (DTP), renal cancer cells and diffuse large B-cell lymphoma (DLBCL) are more sensitive to erastin and more susceptible to ferroptosis than cancer cells from the other six tissues (central nervous system, lung, breast, ovary, melanocytes, and colon) (Xie et al., 2016). In many cases, ferroptosis inducers may be beneficial, and the combination of the ferroptosis inducer erastin with certain chemotherapeutic agents (cisplatin, doxorubicin/adriamycin, cytarabine/ara-C and temozolomide) enhances its antitumour activity and has a favourable prognosis in patients treated with chemotherapy alone (Chen et al., 2015b; Yu et al., 2015). In terms of specific tumour therapy, the System xc-inhibitor imidazole-ketone-erastin (IKE) was effective in xenografts of DLBCL, and it upregulated ferroptosis markers in xenografts (Zhang et al., 2019b). Ovarian cancer is a common and deadly gynecological cancer (Sung et al., 2021). Among them, high-grade serous ovarian cancer (HGSOC) is the most common subtype, with a low 5-year survival rate of only 9–34% (Vang et al., 2009). Although clinically relevant treatments have certain curative effects, most patients will develop cancer cell metastasis, develop drug resistance, and eventually develop into end-stage cancer. However, ovarian cancer cells are susceptible to ferroptosis due to their tumour-initiating cells (TICs) iron addiction, which reduces the level of the iron efflux pump (FPN) and overexpresses the iron importer (TFR1) (Basuli et al., 2017). Therefore, effective targeting of TIC therapy may improve the difficult problem of ovarian cancer treatment. Some experiments have shown that anaesthetics can induce ferroptosis. Experimental studies by Sun et al. indicated that lidocaine could inhibit SLC7A11 expression and enhance ferroptosis in ovarian cancer cells in a dose-dependent and time-dependent manner. We speculate whether local anaesthetics also have auxiliary functions in treating specific tumours, and further research is needed. Despite of significant advances in tumour therapy, tumour drug resistance remains a serious problem. Viswanathan et al. (Viswanathan et al., 2017)found that the treatment resistance state of cancer cells is related to the lipid peroxidase pathway regulated by GPX4, and ketamine can inhibit GPX4 expression. ACSL4-mediated lipid metabolism is also related to tumour metastasis and invasion. Xinyu Wu et al. found that ACSL4 promoted the invasion and migration of prostate and breast cancer cells (Wu et al., 2013; Wu et al., 2015), while LPO generated by ACSL4-mediated lipid metabolism promoted ferroptosis. This finding suggests that ferroptosis can be used as an effective treatment to inhibit cancer metastasis and invasion, whereas propofol can promote ACSL4 expression, and anaesthetics can also enhance the effect of certain chemotherapeutic drugs, such as propofol enhances the ferroptosis of cervical cancer cells induced by paclitaxel. To sum up, it further shows that the selection of perioperative anaesthetics has a certain significance for the prognosis of cancer. The anti-tumour effect of anaesthetics has once again broadened the range of drugs for clinicians, and opened up a new option for cancer treatment.

### Neurodegeneration

Ferroptosis is also involved in sevoflurane- and isoflurane-induced neurodegenerative diseases. Anaesthesia-related neurodegenerative diseases are mainly POCD, which is particularly severe in elderly patients, manifesting as impairments in memory, concentration, and information processing, as well as changes in personality, which can cause serious problems for patients and caregivers to trouble (Batistaki et al., 2017). In addition, POCD is irreversible in many cases, can persist for years after surgery, and increases the overall morbidity and mortality (Subramaniyan and Terrando, 2019). Fer-1 can significantly reduce learning and memory impairment induced by inhalation anaesthetics, and fer-1 preconditioning may be a potential clinical intervention for neuroprotection (Xia et al., 2018), with significant protection from surgery in paediatric and elderly patients. However, increasing evidence suggests a link between the pathogenesis of AD and POCD (Wang et al., 2022). Iron chelators and fer-1 have been shown to prevent AD development by protecting the stability of HIF1A in the brain, thereby inhibiting neuronal death, including ferroptosis (Tang et al., 2021; Zhang et al., 2018b).

### Ischaemia-reperfusion injury

Rational use of dexmedetomidine, lidocaine, propofol, and etomidate can reduce the expression of ferroptosis and protect organs from IRI. Inflammation and massive cell death are caused by I/R in affected organs, leading to ischaemic heart disease, stroke, intestinal injury, renal failure, *etc.* Afterwards, it was confirmed that ferroptosis was involved in the progression of I/R (Friedmann Angeli et al., 2014; Gao et al., 2015; Li et al., 2019b; Tuo et al., 2017). Remarkably, ischaemic heart disease without effective treatment results in the highest mortality rate worldwide (Benjamin et al., 2017). Although the mechanism of ischaemia-induced cell death is elusive, there are still no therapies that act by preventing ischaemic organ injury (IOI)-related cell death. However, over the past few years, multiple studies have identified ferroptosis as a major contributor to the associated cell death in IOI, highlighting the potential value of ferroptosis-targeted treatment in ischaemic heart disease. This was fully confirmed in an isolated mouse heart model mimicking I/R, as well as in *in vivo* experiments, where pharmacological inhibition of ferroptosis by both glutaminase inhibitors and iron chelators significantly reduced cardiac injury (Fang et al., 2019b; Gao et al., 2015).

### Other pathological conditions

Lung and liver fibrosis (Yu et al., 2020; Zhang et al., 2018c), chronic obstructive pulmonary disease from smoking (Park et al., 2019; Yoshida et al., 2019), tissue necrosis from tuberculosis (Amaral et al., 2019), and a rare genetic disorder called Pelizaeus-Merzbacher disease (Nobuta et al., 2019) have also been linked to ferroptosis. There are still gaps in the research of anaesthetic drugs on these diseases, which can be further explored in the future.

## Conclusion and outlook

Ferroptosis plays an important role in the pathological process of tumours, I/R, ALI, and neurodegenerative disorders. Therefore, research on ferroptosis protection is significant for preventing and treating acute diseases. Anaesthetic drugs protect against ferroptosis or promote tumour cell ferroptosis through the System xc-/GPX4 pathways, iron metabolism, lipid metabolism and other pathways. The choice of anaesthetics is of great significance to postoperative disease outcomes. However, along with increasing exposure time and concentration, sevoflurane, lidocaine, and bupivacaine lead to ferroptosis, while the effect of other anaesthetics on ferroptosis has not been reported and needs to be further explored. Whether different analgesics also affect cell ferroptosis has not yet been reported. The gene profile epigenetic regulation of ferroptosis by anaesthetics also needs to be further studied.

Furthermore, the exact molecular mechanism and pathophysiology of drug-related ferroptosis remain to be further investigated, and some conflicting results remain to be further discussed. For example, at the same clinically relevant concentrations, propofol appears to inhibit ferroptosis in the I/R model, whereas for tumour cells, propofol appears to promote ferroptosis. Summarizing the above and Table 3, we found that in the I/R model, the effect of narcotics is anti-ferroptosis, which protects the body. For cancer cells, the effect of anaesthetic drugs is to promote ferroptosis. This may be due to the different responses of anaesthetics to different disease models and microenvironments. Different models show different effects under different conditions, further evidence is needed in the future. Meanwhile, inflammatory cytokines are often increased in ferroptosis. While some anaesthetics, such as ketamine, are anti-inflammatory, they appear to promote ferroptosis in cancer cells. We found none of the experiments showing that ketamine induces both ferroptosis and anti-inflammatory effects. We speculate that the mechanism of action of anaesthetics varies in different experiments and in different cellular systems. It may exhibit anti-inflammatory and anti-ferroptosis effects in inflammatory diseases such as IRI, which requires further validation. We also found that for the same disease model, different anaesthetics can exhibit the same effect, especially in terms of lung protection. For example, in the ALI disease model, the effects of relevant concentrations of DEX, Sev, and lidocaine were shown to be antiferroptotic. Whether other drugs have similar effects needs to be further expanded in the future. In addition, since the mechanisms underlying the action of different anaesthetics on ferroptosis are not precisely identical, it is necessary to have a deeper understanding of the action of different anaesthetics on ferroptosis to provide a theoretical basis for exploring the potential targets of intraoperative anaesthesia to protect cells from ferroptosis. We propose that studying the interaction of anaesthetics and ferroptosis in humans will be an active area of research in the anaesthesia field in the next few years.

## Glossary

ACSL4: acyl CoA synthase long-chain family member 4
ALI: acute lung injury
ALOX: arachidonic acid lipoxygenase
AMPK: 5′ AMP-activated protein kinase
CLP: caecal ligation and puncture
CPX: ciclopirox
DFO: deferoxamine
DFP: deferiprone
DEX: dexmedetomidine
DLBCL: diffuse large B-cell lymphoma
DMF: dimethyl fumarate
DMT1: divalent metal transporter 1
ETC: electron transport chain
Fer-1: ferrostatin-1
FTH1: ferritin heavy chain-1
GPX4: glutathione peroxidase 4
GSH: glutathione
GSSG: glutathione oxidized
HO-1: haem oxygenase-1
H3K27ac: histone H3 lysine 27 acetylation
H/R: hypoxia/reoxygenation
IKE: imidazole-ketone-erastin
IOI: ischaemic organ injury
I/R: ischaemia/reperfusion
IRI: ischaemia-reperfusion injury
IRP2: iron regulatory proteins 2
L-bupivacaine: levobupivacaine
Lip-1: liproxstatin-1
LPCAT3: lysophosphatidylcholine acyltransferase 3
LPS: lipopolysaccharide
MDA: malondialdehyde
MDM2: murine double minute 2
MIRI: myocardial ischaemia reperfusion injury
mTOR: mammalian target of rapamycin
NMDAR: excitatory N-methyl-D-aspartate receptors
Nrf2: nuclear factor erythroid 2-related factor 2
NSCLC: Non-small cell lung cancer
POCD: postoperative cognitive dysfunction
PL-PUFA: phospholipid-bound polyunsaturated fatty acids
PUFA: polyunsaturated fatty acids
PPP: pentose phosphate pathway
ROS: reactive oxygen species
Sev: sevoflurane
SLC7A11: solute carrier family 7 members 11
TFR1: transferrin receptor 1
VECs: vascular endothelial cells
2,2-BP: 2,2-bipyridile
